# NeuroFANN: identification of neuropathological subtypes in dementia with plasma proteins by using functionally annotated neural network

**DOI:** 10.1093/bib/bbaf366

**Published:** 2025-08-01

**Authors:** Sunghong Park, Doyoon Kim, Ji-Hye Choi, Chang Hyung Hong, Sang Joon Son, Hyun Woong Roh, Hyunjung Shin, Hyun Goo Woo

**Affiliations:** Department of Physiology, Ajou University School of Medicine, Worldcup-ro 164, Yeongtong-gu, Suwon, 16499, Republic of Korea; Department of Physiology, Ajou University School of Medicine, Worldcup-ro 164, Yeongtong-gu, Suwon, 16499, Republic of Korea; Department of Physiology, Ajou University School of Medicine, Worldcup-ro 164, Yeongtong-gu, Suwon, 16499, Republic of Korea; Department of Psychiatry, Ajou University School of Medicine, Worldcup-ro 164, Yeongtong-gu, Suwon, 16499, Republic of Korea; Department of Psychiatry, Ajou University School of Medicine, Worldcup-ro 164, Yeongtong-gu, Suwon, 16499, Republic of Korea; Department of Psychiatry, Ajou University School of Medicine, Worldcup-ro 164, Yeongtong-gu, Suwon, 16499, Republic of Korea; Department of Industrial Engineering, Ajou University, Worldcup-ro 206, Yeongtong-gu, Suwon, 16499, Republic of Korea; Department of Artificial Intelligence, Ajou University, Worldcup-ro 206, Yeongtong-gu, Suwon, 16499, Republic of Korea; Department of Physiology, Ajou University School of Medicine, Worldcup-ro 164, Yeongtong-gu, Suwon, 16499, Republic of Korea; Department of Biomedical Science, Graduate School of Ajou University, Worldcup-ro 164, Yeongtong-gu, Suwon, 16499, Republic of Korea; Ajou Translational Omics Center, Research Institute for Innovative Medicine, Ajou University Medical Center, Worldcup-ro 164, Yeongtong-gu, Suwon, 16499, Republic of Korea

**Keywords:** dementia, neuropathological subtype, plasma proteomic profile, protein–protein interaction, biologically functional annotation, graph-based machine learning

## Abstract

Dementia diagnosis relies on identifying neuropathological features, such as beta-amyloid (Aβ) deposition, medial temporal lobe atrophy (MTA), and white matter hyperintensity (WMH). Recently, plasma protein biomarkers have emerged as a cost-effective and less invasive tool for identifying neuropathological features, enhanced by machine learning (ML) for precise diagnosis. However, most ML studies fail to account for protein–protein interactions (PPIs) and synergetic effects between proteins, overlooking their collective contributions to disease mechanisms. Additionally, the lack of consideration for functional properties may result in the redundant and imbalanced representation of proteins and their functions, potentially limiting the effectiveness of dementia diagnosis. In this study, we propose NeuroFANN, a method designed to classify three neuropathological subtypes in dementia—positivity for Aβ, MTA, and WMH—using plasma protein biomarkers. A key feature of NeuroFANN is the combination of the PPI network-based synergetic effects with the functional annotation-based protein biomarker clustering. NeuroFANN extracts synergetic effects by propagating independent effects of proteins across the PPI network, which are then aggregated in functional protein clusters, thereby enabling global PPI awareness and capturing the biological properties of protein biomarkers. From a South Korean cohort, 54 proteins were identified as plasma protein biomarkers for dementia subtypes and grouped into 16 clusters. NeuroFANN outperformed comparison methods in classifying dementia subtypes, with its core components validated as key contributors to superior performance. Additionally, the risk scores predicted by NeuroFANN showed a strong association with longitudinal cognitive decline, demonstrating its potential as a valuable diagnostic tool in clinical settings.

## Introduction

Dementia is a disease of degenerative brain changes characterized by cognitive decline and impairment in daily living functions [1]. Several subtypes of dementia have been identified, including the most common type of Alzheimer's disease (AD) and vascular dementia [2], each associated with distinct neuropathological mechanisms [2]. Key pathological features include amyloid-beta (Aβ) accumulation, tau protein deposition, medial temporal lobe atrophy (MTA), and white matter hyperintensity (WMH) [3–5], all of which are closely associated with clinical symptoms and patient prognosis [6, 7].

Specifically, Aβ pathology, a hallmark of AD, involves the aggregation of Aβ proteins and subsequent plaque formation in the brain [8]. MTA pathology represents structural degeneration resulting primarily from neuronal injury, synaptic loss, and neuroinflammation, particularly within the hippocampus and medial temporal lobes. This makes MTA a crucial biomarker for diagnosing AD and distinguishing it from other dementia subtypes [9]. WMH pathology arises due to small vessel disease and is characterized by endothelial dysfunction, chronic vascular inflammation, and blood–brain barrier disruption [10]. These neuropathological features align well with established frameworks such as ATN (amyloid, tau, neurodegeneration) and vascular pathology [11, 12], playing critical roles in the classification of dementia subtypes and informing clinical management strategies.

Conventional diagnostic approaches for identifying neuropathological features include neuroimaging and cerebrospinal fluid (CSF) biomarkers. Positron emission tomography (PET), for instance, is used to detect Aβ deposition, while magnetic resonance imaging (MRI) assesses the severity of MTA [13]. Additionally, CSF biomarkers, such as Aβ42 and phosphorylated tau proteins, are widely utilized [14, 15]. However, PET and MRI are costly and have limited accessibility, and CSF testing is invasive with the potential for serious complications. Moreover, both methods have limitations in fully capturing the complex molecular pathology of dementia.

Recent advancements in neurology have highlighted plasma protein biomarkers as promising alternatives for diagnosing various neurodegenerative diseases and understanding their underlying mechanisms. Plasma biomarkers offer advantages over CSF biomarkers and neuroimaging by being less invasive and more cost-effective [16, 17]. Integrating these biomarkers into existing medical systems could reduce patient burden and improve diagnostic accessibility. Consequently, plasma biomarkers have gained increasing adoption across various disorders beyond neurodegenerative diseases [18, 19].

Plasma protein biomarkers effectively reflect distinct dementia pathologies at the molecular level, including Aβ, MTA, and WMH. First, proteins involved in amyloid processing and clearance, such as apolipoprotein E, complement, and inflammation-related proteins, significantly correlate with brain amyloid deposition [20–22]. Second, plasma neurofilament light chain, indicative of neuronal injury, and glial fibrillary acidic protein, a marker of neuroinflammation, reliably reflect MTA pathology [23–25]. Third, plasma proteins indicative of endothelial dysfunction, vascular inflammation, and blood–brain barrier integrity have been extensively linked to white matter lesion burden, thereby biologically reflecting cerebrovascular injury associated with WMH [26–28]. These molecular associations enhance our understanding of dementia pathogenesis and improve diagnostic accuracy, particularly in early disease stages.

Furthermore, the integration of machine learning (ML) has significantly advanced plasma biomarker-based diagnostic approaches, providing deeper insights into dementia and other diseases [29, 30]. However, existing ML studies on dementia biomarkers often emphasize only the independent effects of individual proteins, overlooking their synergetic interactions within biological networks [31–33]. This narrow approach contrasts with broader trends observed in other omics-based research fields—such as cancer subtype prediction, multi-omics drug-response modeling, and rare-disease gene discovery—where network-based aggregation of PPIs has become a standard analytical practice [34–36]. Similarly, plasma biomarker-based dementia studies have rarely utilized functional annotations guided feature grouping methods [37, 38], despite their widespread use in transcriptomic and proteomic analyses to elucidate underlying biological processes and ensure balanced functional representation [39, 40]. Neglecting these strategies may lead to redundancy among selected biomarkers and imbalanced representations of biological functions [41], ultimately compromising the effectiveness and interpretability of diagnostic models.

To address these challenges, the present study aims to develop a novel ML model based on a graph neural network (GNN), namely NeuroFANN, for predicting three neuropathological subtypes in dementia—positivity for Aβ, MTA, and WMH—by employing plasma biomarkers. A distinguishing feature of NeuroFANN is its utilization of the PPI network and functional annotations for plasma biomarkers. This strategy enables the extraction of the synergetic effects between biomarkers, capturing the collective contribution of multiple proteins to distinct dementia subtypes. Furthermore, it facilitates a functionally balanced analysis informed by understanding the underlying biological processes.

## Methods

### Overview of the study

**Figure 1:**
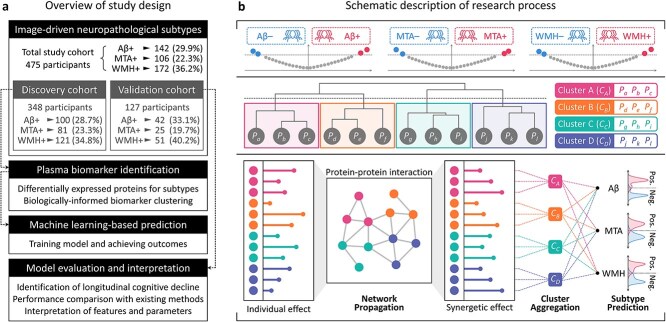
Overview of the study. (a) Represents the overview of the study design. The proposed method begins by identifying plasma biomarkers associated with neuropathological subtypes in dementia using differential expression analysis. These biomarkers are then grouped based on their functional annotations. Individual risks for dementia subtypes are subsequently predicted using NeuroFANN. (b) Schematically describes the proposed method. NeuroFANN propagates the independent effects of biomarkers onto the PPI network, extracting synergetic effects that account for global interactions between proteins. These synergetic effects are aggregated within predefined clusters, aligning with the functional annotations of the plasma biomarkers.

As shown in Fig. 1a, this study begins with the identification of plasma proteins associated with neuropathological subtypes of dementia through differential expression analysis (DEA), followed by the clustering of these protein biomarkers based on their functional annotations. Next, NeuroFANN is used to predict individual risks for dementia subtypes. As illustrated in Fig. 1b, the model projects the independent effects of proteins that passed the DEA criteria onto the PPI network, capturing synergetic effects that reflect the global properties of PPIs. The synergetic effects are then aggregated within predefined clusters, reflecting the functional annotations of plasma protein biomarkers. The key contributions of this study are summarized below.

- We propose a novel GNN-based method, NeuroFANN, for identifying neuropathological subtypes in dementia with plasma proteins.
- NeuroFANN extracts the synergetic effects between proteins through the PPI network, capturing the collective impact of multiple proteins on the dementia subtypes.
- NeuroFANN achieves improved outcomes by utilizing biologically informed clustering based on the functional annotations of plasma protein biomarkers.

### Differential expression analysis

The proposed method initially identifies differentially expressed proteins (DEPs) for neuropathological subtypes of dementia. The expression levels of each protein are compared between the positive and negative groups for each subtype, and the statistical significance of those differences is subsequently analyzed. To carry out this analysis, the “limma” R/Bioconductor package [42] is utilized. Consequently, a union of proteins that are differentially expressed for the three subtypes is employed as plasma protein biomarkers among a total of assayed proteins.

### Biologically informed protein clustering

Biologically informed protein clustering comprises two steps: functional grouping and biomarker clustering. This process begins with identifying Gene Ontology (GO) groups consisting of GO terms significantly associated with the plasma biomarkers. Subsequently, a hierarchical clustering analysis is performed based on the incidence matrix for the GO groups and their corresponding plasma biomarkers.

#### Functional grouping

The proposed method firstly identifies the functional annotations for the plasma biomarkers by using the “‘Functional Annotation Clustering” tool, which is provided by the Database for Annotation, Visualization, and Integrated Discovery (DAVID version 2021 with DAVID Knowledgebase version 2024q2, https://david.ncifcrf.gov). The running options are as follows: similarity term overlap = 3, similarity threshold = 1, initial group membership = 2, final group membership = 2, multiple linkage threshold = 1, and enrichment thresholds = 0.05. The resulting outcomes are represented as the binary incidence matrix for the GO groups and their corresponding plasma protein biomarkers.

#### Biomarker clustering

The proposed method subsequently conducts a hierarchical clustering analysis on the protein biomarkers. The distances between proteins are calculated by the incidence matrix from the functional grouping using the Jaccard metric. The distances between clusters are measured using Ward’s method, and the cutoff is set as a minimum value that ensures all clusters contain a minimum of two biomarkers.

### Functionally annotated neural network

#### Model implementation

NeuroFANN consists of three main components: network propagation, cluster aggregation, and subtype prediction. First, the independent effects of plasma biomarkers are propagated through a PPI network to capture the synergetic effects among biomarkers. Next, the synergetic effects of biomarkers within the same cluster are aggregated, with each biomarker’s contribution modulated by a trainable importance parameter. Finally, these aggregated cluster-level effects are used to predict risks of neuropathological subtypes.

##### Network propagation

Let $p$ and $n$ denote the numbers of plasma biomarkers and study participants, respectively. We define $\mathbf{X}\in{\mathbb{R}}^{p\times n}$ as the matrix of independent effect, and $\mathbf{W}\in{\mathbb{R}}^{p\times p}$ as the matrix representing the PPI network. We aim to derive a propagated signal matrix $\mathbf{H}\in{\mathbb{R}}^{p\times n}$ capturing the synergetic effects by propagating $\mathbf{X}$ through $\mathbf{W}$. Formally, we address the following optimization problem:


(1) \begin{equation*} \underset{\mathbf{H}}{\min}\mathrm{Tr}\left({\mathbf{H}}^{\mathrm{T}}\mathbf{LH}\right)+\mathrm{Tr}\left({\left(\mathbf{H}-\mathbf{X}\right)}^{\mathrm{T}}\boldsymbol{\Phi} \left(\mathbf{H}-\mathbf{X}\right)\right) \end{equation*}

where $\mathbf{L}$ denotes the normalized graph Laplacian of $\mathbf{W}$, defined as $\mathbf{L}={\mathbf{I}}_p-{\mathbf{D}}^{-1/2}\mathbf{W}{\mathbf{D}}^{-1/2}\ \left({\mathbf{W}}^{\mathrm{T}}=\mathbf{W}\ge \mathbf{0}\right)$, with $\mathbf{D}$ being a diagonal matrix whose elements are ${\mathbf{D}}^{\left(i,i\right)}={\sum}_k{\mathbf{W}}^{\left(i,k\right)}$. $\boldsymbol{\Phi} \in{\mathbb{R}}^{p\times p}$ is a diagonal matrix with diagonal entries $\boldsymbol{\phi}=\left({{\phi}}^{(1)},\cdots,{{\phi}}^{(p)}\right) \in{\mathbb{R}}^{p}$ ($\boldsymbol{\Phi} =\operatorname{Diag}\left(\boldsymbol{\phi} \right)>\mathbf{0}$), balancing the smoothness term ($\mathrm{Tr}\left({\mathbf{H}}^{\mathrm{T}}\mathbf{LH}\right)$) against the steadiness term ($\mathrm{Tr}\left({\left(\mathbf{H}-\mathbf{X}\right)}^{\mathrm{T}}\boldsymbol{\Phi} \left(\mathbf{H}-\mathbf{X}\right)\right)$). Biologically, the smoothness term ensures minimal differences in propagated signals between adjacent proteins within the PPI network, emphasizing coordinated actions and functional coherence among interacting proteins. Conversely, the steadiness term preserves original individual biomarker signals, preventing excessive dilution of protein-specific effects during network propagation. The closed-form solution to this optimization problem is given by:


$$ \mathbf{H}={\left(\boldsymbol{\Phi} +\mathbf{L}\right)}^{-1}\boldsymbol{\Phi} \mathbf{X}. $$

##### Cluster aggregation

The derived synergetic effects in $ \mathbf{H} $ are then aggregated at the cluster level to produce $\mathbf{Z}=\left\{{\mathbf{Z}}_1;\cdots; {\mathbf{Z}}_k;\cdots; {\mathbf{Z}}_m\right\} \in{\mathbb{R}}^{m\times n}$, where $ m $ is the number of biomarker clusters. For the $ k $-th cluster $\mathbf{C}_k$, its aggregated effect $ {\mathbf{Z}}_k $ is:


$${\mathbf{Z}}_k=\sum_{i\in{C}_k}{\boldsymbol{\Lambda}}_k^{(i)}{\mathbf{H}}_k^{(i)}, \textrm{where}\ {\boldsymbol{\Lambda}}_k^{(i)}={e}^{{\boldsymbol{\lambda}}_k^{(i)}}/{\sum}_j{e}^{{\boldsymbol{\lambda}}_k^{(j)}}.$$

Here, ${\mathbf{H}}_k$ denotes the subset of $\mathbf{H}$ corresponding to biomarkers within cluster ${\mathbf{C}}_k$, and ${\boldsymbol{\lambda}}_k=\left({\boldsymbol{\lambda}}_k^{(1)},\cdots, {\boldsymbol{\lambda}}_k^{\left({p}_k\right)}\right)$ indicates the trainable importance parameter vector for ${\mathbf{C}}_k$. The parameters ${\boldsymbol{\lambda}}_k^{\left(\ast \right)}$ are initialized with uniform weights inversely proportional to their cluster size, specifically $1/\left|{\mathbf{C}}_k\right|$. After training, the optimized ${\boldsymbol{\lambda}}_k^{\left(\ast \right)}$ values represent the relative importance of proteins within ${\mathbf{C}}_k$. The exponentiated terms ${e}^{{\boldsymbol{\lambda}}_k^{\left(\ast \right)}}$ normalize to ensure the contribution weights within each cluster sum to 1. Consequently, higher ${\boldsymbol{\lambda}}_k^{\left(\ast \right)}$ values indicate that the corresponding proteins contribute more significantly to the functional role of ${\mathbf{C}}_k$ in relation to neuropathological subtypes, compared to other proteins in the same cluster.

##### Subtype prediction

Finally, NeuroFANN predicts the risk for each neuropathological subtype based on the aggregated cluster effects. Denote ${\boldsymbol{\Theta}}_{\ast}\in{\mathbb{R}}^m$ by the prediction parameters for a subtype $\ast \in\left\{\mathrm{A\beta},\ \mathrm{MTA},\ \mathrm{WMH}\right\}$. The predicted risk vector ${\mathbf{P}}_{\ast} \in{\mathbb{R}}^n$ is obtained via a logistic function:


$$ {\mathbf{P}}_{\ast }=\frac{1}{1+{e}^{-{{\boldsymbol{\Theta}}_{\ast}}^{\mathrm{T}}\mathbf{Z}}}. $$

#### Parameter optimization

NeuroFANN jointly optimizes three parameter sets: the propagation parameter ($\boldsymbol{\phi}$) for network propagation, the importance parameter ($\boldsymbol{\lambda}$) for cluster aggregation, and the prediction parameter (${\boldsymbol{\Theta}}_{\ast }$) for subtype prediction. These parameters are trained to minimize the binary cross-entropy:


$$ {\mathcal{L}}_{\ast }=-\frac{1}{n}\left\{\left({{\mathbf{Y}}_{\ast}}^{\mathrm{T}}\log{\mathbf{P}}_{\ast}\right)+{\left({\mathbf{1}}_n-{\mathbf{Y}}_{\ast}\right)}^{\mathrm{T}}\log \left({\mathbf{1}}_n-{\mathbf{P}}_{\ast}\right)\right\}, $$

where ${\mathbf{Y}}_{\ast} \in{\left\{0,1\right\}}^n$ indicates subtype presence. The overall objective, including regularization, is:


(2) \begin{equation*} \underset{\boldsymbol{\phi},\boldsymbol{\lambda}, {\boldsymbol{\Theta}}_{\ast }}{\mathrm{argmin}}\kern0.5em \sum{\mathcal{L}}_{\ast }+\delta \mathcal{R} \end{equation*}

where $\mathcal{R}$ is the ${\ell}_2$ regularization term defined as $\mathcal{R}={\left\Vert \boldsymbol{\phi} \right\Vert}_2^2+{\left\Vert \boldsymbol{\lambda} \right\Vert}_2^2+{\left\Vert{\boldsymbol{\Theta}}_{\mathrm{A}\mathrm{\beta}}\right\Vert}_2^2+{\left\Vert{\boldsymbol{\Theta}}_{\mathrm{MTA}}\right\Vert}_2^2+{\left\Vert{\boldsymbol{\Theta}}_{\mathrm{WMH}}\right\Vert}_2^2$, and $\delta$ is the regularization coefficient. The objective function in equation (2) is optimized via gradient descent method [43], updating each parameter set in turn.

Minimization over ${\Theta}_{\ast }$: For each subtype $\ast$, we first differentiate the terms in equation (2) as:


$$ \frac{\partial{\mathcal{L}}_{\ast }}{\partial{\boldsymbol{\Theta}}_{\ast }}=\frac{1}{n}\mathbf{Z}{\left({\mathbf{P}}_{\ast }-{\mathbf{Y}}_{\ast}\right)}^{\mathrm{T}},\kern0.5em \frac{\partial \mathcal{R}}{\partial{\boldsymbol{\Theta}}_{\ast }}=2\delta{\boldsymbol{\Theta}}_{\ast }. $$

Combining these terms, the gradient with respect to $ {\boldsymbol{\Theta}}_{\ast } $ becomes:


$$ \nabla{\boldsymbol{\Theta}}_{\ast }=\frac{1}{n}\mathbf{Z}{\left({\mathbf{P}}_{\ast }-{\mathbf{Y}}_{\ast}\right)}^{\mathrm{T}}+2\delta{\boldsymbol{\Theta}}_{\ast }. $$

Minimization over $\lambda$: To find the gradient with respect to the importance parameter $\boldsymbol{\lambda}$, we first derive $\partial \mathcal{L}/\partial \mathbf{Z}$ as below:


$$ \frac{\partial \mathcal{L}}{\partial \mathbf{Z}}=\sum \frac{\partial{\mathcal{L}}_{\ast }}{\partial \mathbf{Z}}=\sum \frac{1}{n}{\boldsymbol{\Theta}}_{\ast}\left({\mathbf{P}}_{\ast }-{\mathbf{Y}}_{\ast}\right)\mathbf{\in}{\mathbb{R}}^{m\times n}. $$

By partitioning $\partial \mathcal{L}/\partial \mathbf{Z}$ as $\partial \mathcal{L}/\partial \mathbf{Z}=\big[\partial \mathcal{L}/\partial{\mathbf{Z}}_1;\cdots; \partial \mathcal{L}/\partial{\mathbf{Z}}_k;\cdots; \partial \mathcal{L}/\partial\\{\mathbf{Z}}_m\big]$, we compute $\partial \mathcal{L}/\partial{\boldsymbol{\lambda}}_k$ by combining $\partial \mathcal{L}/\partial{\mathbf{Z}}_k$ with $\partial{\mathbf{Z}}_k/\partial{\boldsymbol{\lambda}}_k$. Consequently, the gradient with respect to ${\boldsymbol{\lambda}}_k$ (the parameter vector for cluster $k$) obtained as follows.


$$ \nabla{\boldsymbol{\lambda}}_k=\frac{\partial \mathcal{L}}{\partial{\mathbf{Z}}_k}{\mathbf{H}}_k^{\mathrm{T}}\left\{\mathrm{Diag}\left({\boldsymbol{\Lambda}}_k\right)-{\boldsymbol{\Lambda}}_k{\boldsymbol{\Lambda}}_k^{\mathrm{T}}\right\}+2\delta{\boldsymbol{\lambda}}_k. $$

Minimization over $\phi$: Lastly, for the propagation parameter $\boldsymbol{\phi}$, we first derive:


$$ \frac{\partial \mathcal{L}}{\partial \mathbf{H}}=\left[\frac{\partial \mathcal{L}}{\partial{\mathbf{H}}_1};\cdots; \frac{\partial \mathcal{L}}{\partial{\mathbf{H}}_k};\cdots; \frac{\partial \mathcal{L}}{\partial{\mathbf{H}}_m}\right],\kern0.5em \frac{\partial \mathcal{L}}{\partial{\mathbf{H}}_k}={\boldsymbol{\lambda}}_k^{\mathrm{T}}\frac{\partial \mathcal{L}}{\partial{\mathbf{Z}}_k}. $$

Combining $ \partial \mathcal{L}/\partial \mathbf{H} $ with $ \partial \mathbf{H}/\partial \boldsymbol{\Phi} $ gives:


$$ \frac{\partial \mathcal{L}}{\partial \boldsymbol{\Phi}}=\frac{\partial \mathcal{L}}{\partial \mathbf{H}}{\left[{\left(\boldsymbol{\Phi} +\mathbf{L}\right)}^{-1}\left\{{\mathbf{I}}_p-{\left(\boldsymbol{\Phi} +\mathbf{L}\right)}^{-1}\boldsymbol{\Phi} \right\}\mathbf{X}\right]}^{\mathrm{T}}. $$

Since $\boldsymbol{\Phi}$ is diagonal, the gradient with respect to $\boldsymbol{\phi}$ (its diagonal entries) is:


$$ \nabla \boldsymbol{\phi} =\operatorname{diag}\left(\frac{\partial \mathcal{L}}{\partial \mathbf{H}}{\left[{\left(\boldsymbol{\Phi} +\mathbf{L}\right)}^{-1}\left\{{\mathbf{I}}_p-{\left(\boldsymbol{\Phi} +\mathbf{L}\right)}^{-1}\boldsymbol{\Phi} \right\}\mathbf{X}\right]}^{\mathrm{T}}\right)+2\delta \boldsymbol{\phi} . $$

### Statistical analysis

We used MATLAB (version R2024a), Python (version 3.9.7), and R (version 3.6.1) for all analyses in this study. Continuous variables are presented as mean with standard deviation (SD) or median with interquartile range, with group-wise comparison using a one-way analysis of variance for Gaussian distribution or the Kruskal–Wallis test for non-Gaussian distribution. Categorical variables are presented as count (percentage), with group-wise comparison using the chi-squared test. Statistical significance was indicated by a two-sided *P*-value.

## Materials

### Study participants

We recruited participants from the Biobank Innovations for chronic Cerebrovascular disease With ALZheimer’s disease Study at Ajou University Hospital (Suwon, Republic of Korea) [44]. The recruited study participants underwent three-dimensional T1-weighted MRI using a 3.0 Tesla scanner and ^18^F-flutemetamol PET scan using a Discovery STE/690 PET/CT scanner. An average of 185 MBq of ^18^F-flutemetamol was intravenously injected as a bolus via the antecubital vein. After a 90-minute uptake period, PET scans were acquired as four consecutive 5-minute frames, totaling 20 minutes. All neuroimaging data were independently reviewed by neuroradiologists.

Subsequently, clinicians determined the neuropathological subtypes of the participants based on MRI and PET scans. Participants were classified as amyloid-positive if their global cortical standardized uptake value ratio exceeded 0.634, a cutoff previously established in elderly Koreans [45]. MTA positivity was assessed using the Scheltens’ scale [46], which grades atrophy severity from 0 to 4 in each hemisphere; participants were classified as MTA-positive if the combined score from both hemispheres was greater than 4. WMH was classified into three severity categories (mild, moderate, and severe) using the Fazekas scale [47]; participants identified with moderate or severe WMH were considered WMH-positive.

In total, among the 475 participants included in the study cohort, 142 (29.9%), 106 (22.3%), and 172 (36.2%) individuals were identified as positive for Aβ, MTA, and WMH, respectively, while 181 (38.1%) participants were negative for all three subtypes. Importantly, these three neuropathological subtypes were not mutually exclusive, reflecting common clinical observations of multiple concurrent pathologies within individuals. Specifically, 104 (21.9%) participants exhibited positivity for two or more subtypes: 82 (17.3%) participants were positive for exactly two subtypes—23 (4.8%) for both Aβ and MTA, 24 (5.1%) for both Aβ and WMH, and 35 (7.4%) for both MTA and WMH. Additionally, 22 (4.6%) participants were classified as positive for all three subtypes.

Finally, we divided the participants into discovery and validation cohorts. The validation cohort comprised participants who underwent cognitive assessments at the two-year follow-up, whereas participants with only baseline assessments were allocated to the discovery cohort. Detailed demographic and clinical characteristics of the study participants are summarized in Table 1.

**Table 1:** Demographic and clinical characteristics of participants

Characteristics	Total participants (*N* = 475)	Discovery cohort (*N* = 348)	Validation cohort (*N* = 127)	*P*-value^a^
Age, median (IQR), yr	73 (67–77)	72 (67–77)	73 (67–78)	.6343
Female, No. (%)	332 (69.9)	241 (69.3)	91 (71.7)	.6146
MMSE, median (IQR)	24 (20–27)	24 (20–27)	24 (22–27)	.0525
CDR-SB, median (IQR)	2.5 (1.5–4.0)	2.5 (1.5–4.5)	2.0 (1.5–3.0)	.0921
GDS, median (IQR)	3 (3–4)	3 (3–4)	3 (3–4)	.2841
APOE *ε*2 carrier, No. (%)	68 (14.3)	48 (13.8)	20 (15.7)	.5912
APOE *ε*4 carrier, No. (%)	129 (27.2)	91 (26.1)	38 (29.9)	.4144
Aβ positivity, No. (%)	142 (29.9)	100 (28.7)	42 (33.1)	.3621
MTA positivity^b^, No. (%)	106 (22.3)	81 (23.3)	25 (19.7)	.4065
WMH positivity^c^, No. (%)	172 (36.2)	121 (34.8)	51 (40.2)	.2806
Only Aβ positivity, No. (%)	73 (15.4)	53 (15.2)	20 (15.7)	.8901
Only MTA positivity, No. (%)	26 (5.5)	20 (5.7)	6 (4.7)	.6653
Only WMH positivity, No. (%)	91 (19.2)	67 (19.3)	24 (18.9)	.9308
Aβ and MTA positivity, No. (%)	23 (4.8)	18 (5.2)	5 (3.9)	.5797
Aβ and WMH positivity, No. (%)	24 (5.1)	11 (3.2)	13 (10.2)	.0018
MTA and WMH positivity, No. (%)	35 (7.4)	25 (7.2)	10 (7.9)	.7994
Aβ, MTA, and WMH positivity, No. (%)	22 (4.6)	18 (5.2)	4 (3.1)	.3543
Aβ, MTA, and WMH negativity, No. (%)	181 (38.1)	136 (39.1)	45 (35.4)	.4698

IQR, interquartile range; MMSE, Mini-Mental Status Examination; CDR-SB, Clinical Dementia Rating Sum of Boxes; GDS, Global Deterioration Scale; APOE, apolipoprotein E; Aβ, amyloid beta; MTA, medial temporal lobe atrophy score; WMH, white matter hyperintensity.

^a^ *P*-values were calculated for each characteristic by means of a comparison between the discovery and validation cohorts.

^b^MTA scale was divided into left and right and subdivided into 0–4 according to severity, and in this study, MTA-positive was set for cases where the sum of left and right sides was five or more.

^c^WMH scale was divided into three types (mild, moderate, and severe), and WMH-positive was set for moderate and severe.

### Proteomic assays

Blood samples from study participants were collected concurrently with neuroimaging and cognitive function assessments. The samples were centrifuged at room temperature at 3000 rpm for 10 minutes to separate plasma and serum supernatants. To ensure high-purity samples, the plasma and serum supernatants underwent an additional centrifugation step under identical conditions, after which the purified supernatants were immediately collected and stored in an ultra-low temperature freezer at −80°C. The plasma samples were profiled by the Olink Target 96 Neurology and Olink Target 48 Cytokine panels. The former comprises 92 established assays associated with neurobiological diseases, while the latter includes 45 selected assays highly relevant to inflammatory processes, more details can be found at: https://olink.com, and the assayed proteins are listed in Supplementary Table S1. The raw data for protein expression underwent quality control (QC) through both internal and external controls in the panels. We excluded proteins with missing frequency over 10% and imputed missing values by using the *k*-nearest neighbor method. Subsequently, the protein expression values were standardized by using *Z*-score normalization and then transformed by the logistic function for scaling.

## Results

### Identified plasma biomarkers and protein clusters

**Figure 2:**
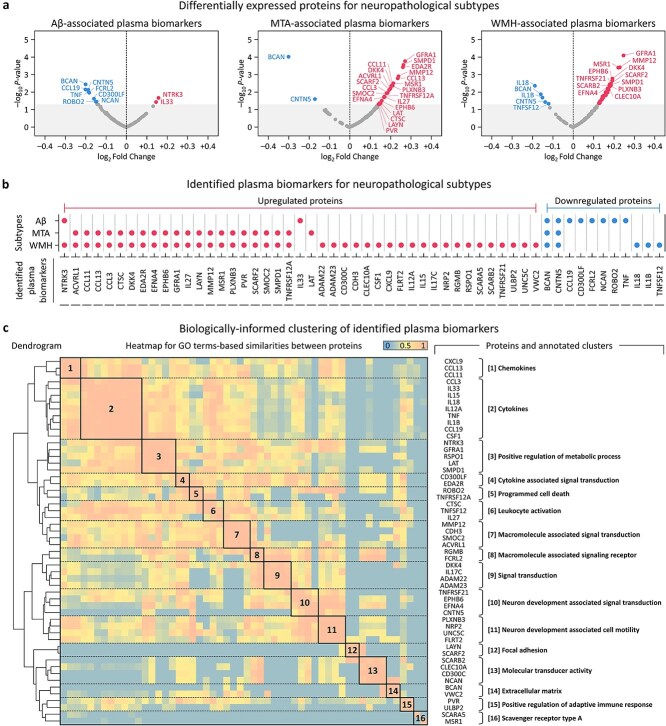
Identified plasma biomarkers and protein clusters for the neuropathological subtypes. (a) Of the 127 proteins that passed quality control process, differential expression analysis identified 10, 23, and 46 proteins as significant plasma biomarkers for aβ, MTA, and WMH, respectively. (b) In total, 54 proteins were identified as plasma biomarkers for three neuropathological subtypes of dementia, comprising 43 upregulated and 11 downregulated proteins. (c) Subsequent clustering analysis grouped the identified biomarkers into 16 clusters.

We conducted DEA for 127 QC-passed proteins out of 137 assayed proteins, identifying 10, 23, and 46 proteins as significant plasma biomarkers for Aβ, MTA, and WMH, respectively (Fig. 2a; Supplementary Table S2). In total, 54 proteins were identified as plasma biomarkers for three neuropathological subtypes, comprising 43 (79.6%) upregulated and 11 (20.4%) downregulated proteins (Fig. 2b). Of those, BCAN and CNTN5 were significantly downregulated proteins in all three subtypes, where those significances associated with dementia have been shown in clinical studies [48, 49], with an average log_2_Fold-Change (FC) of −0.182, which represents a 16.9% reduction compared to an average log_2_FC of −0.156 for the remaining downregulated proteins. NTRK3 was the most significantly upregulated protein for Aβ, demonstrating that NTRK3 signaling is associated with AD by promoting synaptic plasticity and interacting with Aβ-related pathways [50], while GFRA1 indicated the highest degree of upregulation in both MTA and WMH, where GFRA1 has known to bind with the glial cell line-derived neurotrophic factor (GDNF) [51], and the GDNF-GFRA1 complex has revealed to affect hippocampal-related neurological disorders and cerebral white matter damages [52, 53].

Next, we identified 86 GO groups for 239 GO terms that are significantly associated with the plasma biomarkers based on the functional annotation analysis, with each group containing an average of 7.6 (SD, 5.9) biomarkers. By performing clustering analysis, the biomarkers could be grouped into 16 clusters (Fig. 2c; Supplementary Table S3). The “Cytokines” cluster had the highest number of biomarkers (*n* = 9), followed by the ‘Positive regulation of metabolic process’ cluster (*n* = 5), demonstrating the importance of the inflammatory response of cytokines that are related to dementia subtypes based on the association between neurodegeneration and neuroinflammation [54], including the significance of metabolic functions that are associated with dementia [55].

### Predicted outcomes for neuropathological subtypes

**Figure 3:**
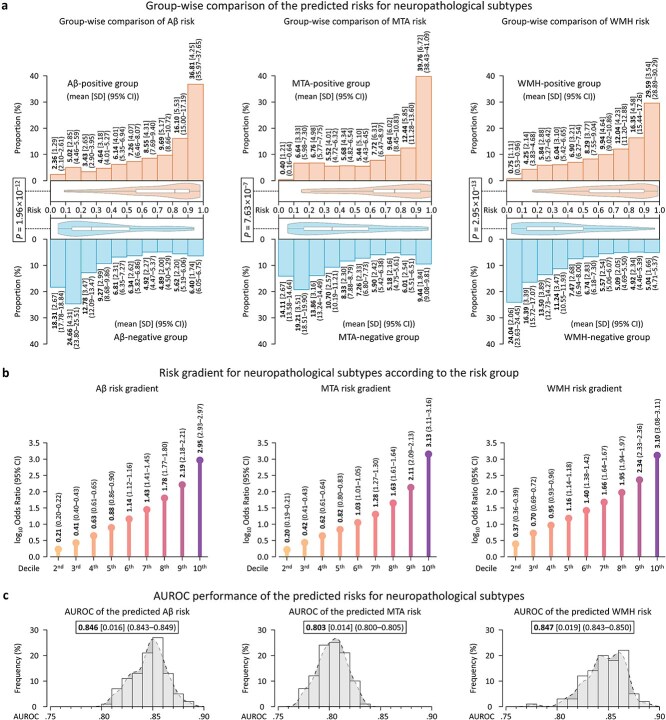
Comparative analyses of predicted outcomes for neuropathological subtypes by NeuroFANN. For each neuropathological dementia subtype, (a) compares the risk-level differences between the diagnostic groups, (b) presents odds ratios according to the risk groups in comparison to the lowest risk group, and (c) evaluates the AUROC performances.

The predicted risks by NeuroFANN demonstrated significant differences between the diagnostic groups for each subtype, with *P*-values of 1.96 × 10^−12^, 7.63 × 10^−7^, and 2.95 × 10^−13^ for Aβ, MTA, and WMH, respectively (Fig. 3a). For the true positive group for each subtype, the proportion of participants in the interval of the highest value in the predicted risk distribution (0.9 or higher) was 35.39% (95% CI, 34.44%–36.33%) on average, with the highest proportion in MTA at 39.76% (95% CI, 38.44%–41.08%), followed by Aβ at 36.81% (95% CI, 35.98%–37.64%) and WMH at 29.59% (95% CI, 28.89%–30.28%). The log_10_odds ratios (log_10_ORs) between the highest and lowest predicted risk groups were 2.95 (95% CI, 2.93%–2.97%), 3.13 (95% CI, 3.11%–3.16%), and 3.10 (95% CI, 3.08%–3.11%) for ABT, MTA, and WMH, respectively, representing an increasing pattern of log_10_ORs in all risk groups across the three subtypes (Fig. 3b). Our model achieved an average AUROC of 0.832 (95% CI, 0.830%–0.833%), where the highest AUROC was observed for WMH at 0.847 (95% CI, 0.843%–0.850%), followed by Aβ at 0.846 (95% CI, 0.843%–0.849%) and MTA at 0.803 (95% CI, 0.800%–0.805%; Fig. 3c).

**Figure 4:**
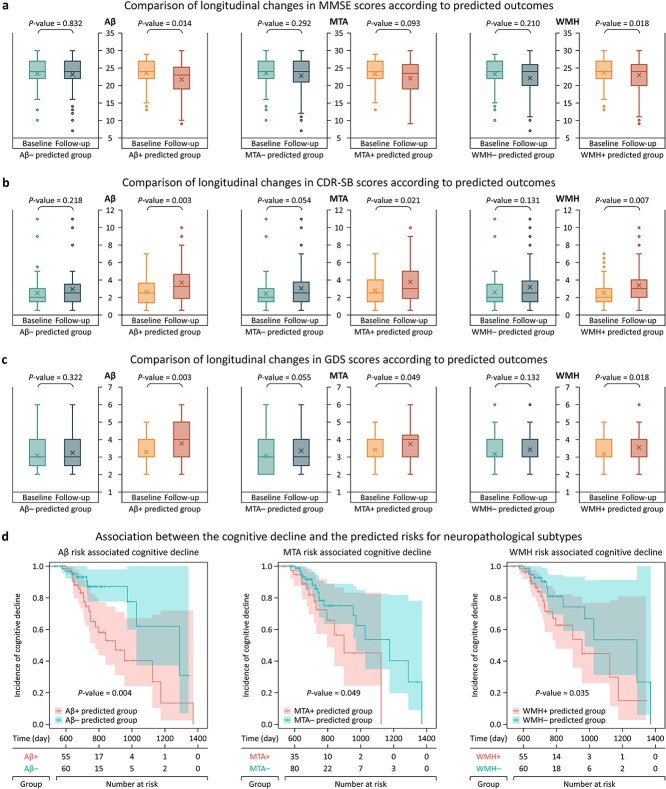
Associations between predicted outcomes and longitudinal changes in cognitive assessments. For patients at risk of each subtype, two-year trajectories were examined for the MMSE (a), CDR-SB (b), and GDS (c). The relationship between cognitive decline and predicted risks was evaluated using survival analysis (d), where cognitive decline was defined as a concurrent deterioration in MMSE, CDR-SB, and GDS scores from baseline to follow-up.

As shown in Fig. 4, we further investigated the longitudinal changes in cognitive function of at-risk patients for each subtype using the MMSE, CDR-SB, and GDS scores assessed over a two-year follow-up. Of the 127 participants in the validation cohort, 58 (45.7%), 42 (33.1%), and 63 (49.6%) were identified as at-risk patients for Aβ, MTA, and WMH, respectively, whose predicted risks for each subtype exceeded the positivity threshold in more than half of the whole predictions. The longitudinal changes for all three test scores in the at-risk patients for Aβ and WMH were statistically significant, and the MTA-at-risk patients indicated significant baseline-to-follow-up differences for CDR-SB and GDS, except MMSE, while the longitudinal changes for all scores in the non-at-risk patients for all subtypes were not statistically significant (Fig. 4a–c). Our investigation also revealed a statistically significant association between the cognitive decline and the predicted risks for all three subtypes. The incidence of cognitive decline was determined by the simultaneous deterioration of MMSE, CDR-SB, and GDS scores from the baseline assessment to the follow-up evaluation (Fig. 4d). The hazard ratios were as follows: Aβ at 2.802 (95% CI, 1.278%–6.143%), MTA at 1.872 (95% CI, 0.880%–3.986%), and WMH at 1.958 (95% CI, 0.937%–4.087%), respectively.

### Model evaluation with performance comparison

#### Experimental settings

Our model was compared with seven GNN-based models: graph convolutional network (GCN) [56], simple graph convolution (SGC) [57], exponential graph convolution (EGC) [58], linear graph convolution (LGC) [58], MixHop [59], universal graph convolutional network (UGCN) [60], and mixed-order graph convolutional network (MOGCN) [61]. The discovery cohort was employed for model training, and the validation cohort was fixed as the test set. The model performance was evaluated by measuring six metrics: area under the receiver operating characteristic curve (AUROC), sensitivity, specificity, positive predictive value (PPV), and negative predictive value (NPV). Each model derived 100 repeated predictions, with 20 iterations of five-fold cross-validation.

#### Performance comparison

**Figure 5:**
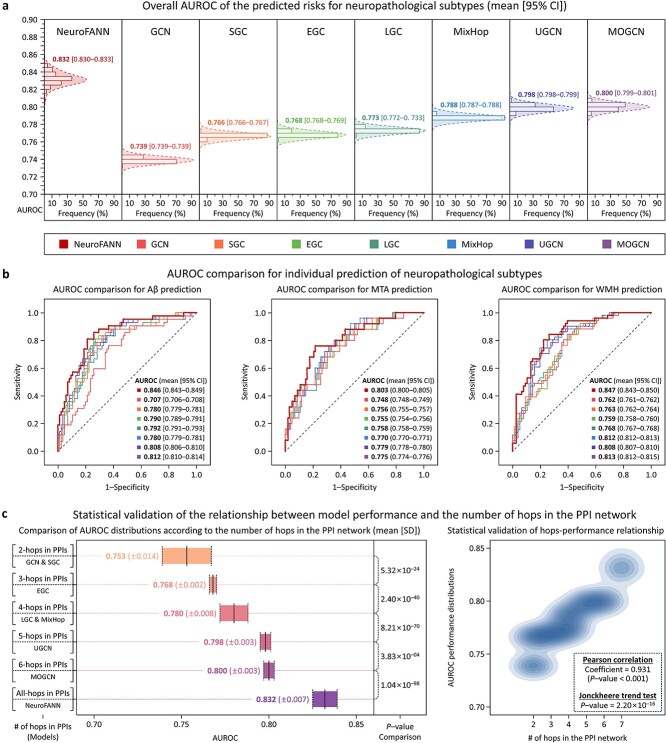
Model evaluation with performance comparison and ablation study. The AUROCs of the ML models were evaluated in terms of their overall performance for the three neuropathological subtypes (a), as well as their individual performance for each subtype (b). The performance of the models was further compared by the number of hops each model reflects in the PPI network, with NeuroFANN defined as having 7 hops for comparability (c).

The performance of NeuroFANN was compared with seven other GNN-based methods (Supplementary Table S4). The average AUROC of the comparison methods was 0.776 (95% CI, 0.775%–0.777%), indicating that our model demonstrated 7.28% (95% CI, 7.06%–7.49%) higher AUROC on average (Fig. 5a). For each subtype, the comparison methods showed average AUROCs as follows: 0.781 (95% CI, 0.779%–0.784%) for Aβ, 0.763 (95% CI, 0.762%–0.764%) for MTA, and 0.784 (95% CI, 0.782%–0.785%) for WMH; thereby demonstrating that our model exhibited improved AUROC for all subtypes: 8.51% (95% CI, 8.11%–8.91%) for Aβ, 5.22% (95% CI, 5.04%–5.40%) for MTA, and 8.17% (95% CI, 7.86%–8.48%) for WMH (Fig. 5b). We further compared the performance of models according to the number of hops each model reflects in the PPI network: 2 hops for GCN and SGC, 3 hops for EGC, 4 hops for LGC and MixHop, 5 hops for UGCN, 6 hops for MOGCN, and all hops for NeuroFANN (defined as 7 hops for comparability). This comparison demonstrated significant performance differences according to the hop count, with a strong correlation between performance and the number of hops (Pearson correlation coefficient = 0.931, *P*–value <0.001; Jonckheere trend test, *P*–value = 2.20 × 10^−16^; Fig. 5c). These findings highlight the importance of utilizing the PPI in a global range; specifically, NeuroFANN’s network propagation effectively captured interactions among distant proteins, while the comparison models only incorporated local interactions between neighboring proteins.

### Empirical analysis of the proposed method

#### Ablation study on key components of NeuroFANN

**Figure 6:**
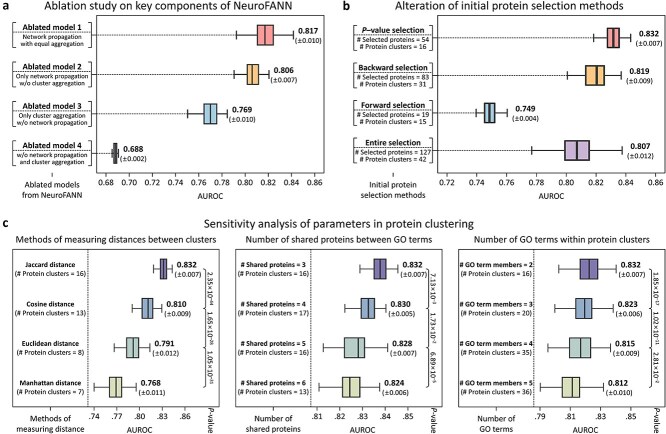
Empirical analysis of the proposed method. First, the contribution of NeuroFANN’s key components—Network propagation and cluster aggregation—To predictive performance was evaluated through four ablated models (a). Next, the impact of different initial protein selection methods on NeuroFANN’s predictive performance was investigated (b). Last, the sensitivity of protein clustering parameters was analyzed (c).

First, we assessed the contributions of NeuroFANN’s key components—network propagation and cluster aggregation—to predictive performance by developing four ablated models (AMs 1–4): AM 1 retained network propagation but employed equal aggregation, where protein clusters were aggregated using uniform weights instead of trained cluster-specific weights; AM 2 excluded the cluster aggregation; AM 3 excluded the network propagation; and AM 4 excluded both network propagation and cluster aggregation. Among these, AM 1 showed the highest AUROC of 0.817 (±0.010), followed by AM 2 at 0.806 (±0.007), and AM 3 at 0.769 (±0.010). AM 4 exhibited the lowest AUROC performance of 0.688 (±0.002). Compared to the average AUROC of these four ablated models, the complete NeuroFANN model achieved an average improvement of 7.65% (Fig. 6a; Supplementary Table S5). These results clearly indicate that the network propagation and cluster aggregation components substantially enhance NeuroFANN’s predictive performance, underscoring their critical roles in effectively integrating PPI information.

#### Comparative evaluation of initial protein selection methods

Next, we evaluated the impact of different initial protein selection strategies on the predictive performance of NeuroFANN. In addition to the univariate DEA-based *P*–value filtering used in this study, multivariate approaches including backward and forward selection methods, as well as an inclusive ‘entire selection’ involving all 127 proteins, were examined. As shown in Fig. 6b, the backward selection identified 83 proteins grouped into 31 clusters, whereas forward selection identified 19 proteins grouped into 15 clusters. In the case of entire selection, all 127 proteins were grouped into 42 clusters. Subsequently, NeuroFANN achieved AUROC values of 0.819, 0.749, and 0.807 for backward, forward, and entire selections, respectively, representing decreases of 1.6%, 11.1%, and 3.1% compared to the baseline DEA-based selection performance of 0.832. These results suggest that a parsimonious feature set derived from DEA-based selection optimally balances informative signal and noise, highlighting the importance of careful biomarker pre-selection in enhancing NeuroFANN’s predictive performance.

#### Sensitivity analysis of protein clustering parameters

Finally, we conducted a sensitivity analysis of protein clustering parameters: (i) inter-cluster distance measures, (ii) the minimum number of shared proteins between GO terms, and (iii) the minimum number of GO terms within protein clusters (Fig. 6c). First, in addition to the baseline Jaccard distance measure, we evaluated Cosine, Euclidean, and Manhattan distances. Compared to the 16 clusters derived by the Jaccard method (AUROC = 0.832), Cosine, Euclidean, and Manhattan distances yielded fewer clusters—13, 8, and 7, respectively—and correspondingly reduced AUROC values (0.810, 0.791, and 0.768, respectively). Second, when increasing the minimum number of shared proteins between GO terms from the original setting of 3 to 4, 5, and 6, the number of clusters changed marginally (16, 17, 16, and 13 clusters, respectively), accompanied by slight decreases in AUROC performance (0.832, 0.830, 0.828, and 0.824, respectively). Third, raising the minimum number of GO terms per cluster from the original setting of 2 to 3, 4, and 5 markedly increased the number of clusters (16, 20, 35, and 36, respectively) while progressively decreasing AUROC performance (0.832, 0.823, 0.815, and 0.812, respectively). Collectively, these findings suggest that NeuroFANN achieves optimal predictive performance under the original, conservatively tuned clustering parameters, highlighting the necessity of biologically balanced parameter settings to preserve informative signals while minimizing performance loss due to either excessive or insufficient aggregation.

### Interpretation of features and parameters

**Figure 7:**
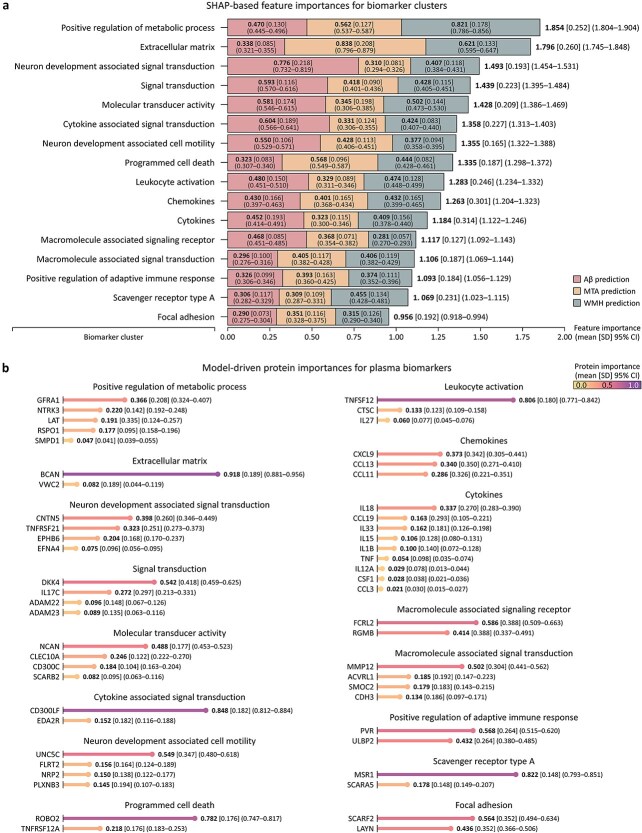
Predictive importance for biomarker clusters and proteins. The lists of SHAP-based feature importance for biomarker clusters (a) and model-driven protein importance for plasma biomarkers (b) are compared.

We evaluated predictive importance of biomarker clusters using SHapley Additive exPlanations (SHAP) [62] (Fig. 7a). The “neuron development associated signal transduction” cluster exhibited the highest importance for Aβ positivity, with a SHAP value of 0.776 (95% CI, 0.732%–0.819%). For MTA and WMH positivity, the most significant clusters were “extracellular matrix” (0.838; 95% CI, 0.796%–0.879%) and “positive regulation of metabolic process” (0.821; 95% CI, 0.786%–0.856%), respectively. These three clusters ranked among the top three overall feature importance, followed closely by clusters such as “signal transduction,” “molecular transducer activity,” “cytokine associated signal transduction,” “neuron development associated cell motility,” and “programmed cell death.” Together, these eight clusters exhibited higher feature importance than the mean value of 1.321 observed across all clusters, identifying them as key clusters for predicting neuropathological subtypes. Subsequently, we analyzed the protein-level importance within the 16 clusters derived from the cluster aggregation step of NeuroFANN (Fig. 7b). In the “positive regulation of metabolic process” cluster, GFRA1 demonstrated the highest protein-level importance (0.366; 95% CI, 0.324%–0.407%), followed by NTRK3 (0.220; 95% CI, 0.192%–0.248%). Additionally, proteins such as BCAN, CNTN, DKK4, NCAN, CD300LF, UNC5C, and ROBO2 showed the greatest importance within their respective clusters. These findings suggest that SHAP-based importance highlight the functional significance of each protein cluster for neuropathological subtypes by capturing their marginal contributions to the model output, while the model-driven importance, determined by the relative weights assigned to individual proteins within clusters, identify proteins that serve as functional representatives within biologically coherent modules. This complementary interpretation underscores the value of our integrated modeling approach, effectively capturing both individual and collective effects of biomarkers.

**Figure 8:**
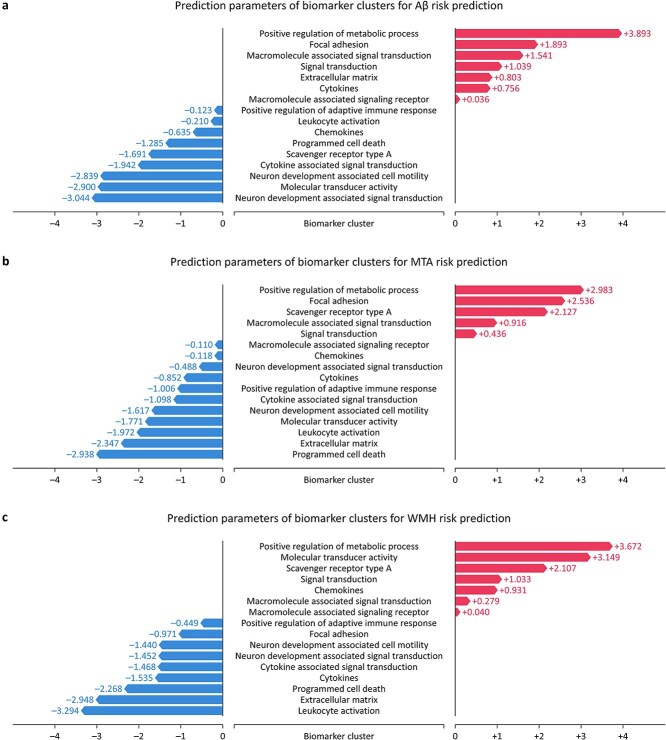
Estimated prediction parameters of biomarker clusters. The prediction parameters of biomarker clusters for each dementia subtype estimated by NeuroFANN were compared.

Lastly, we analyzed the prediction parameters of biomarker clusters (Fig. 8). The clusters with consistently positive prediction parameters across all dementia subtypes were “positive regulation of metabolic process,” “macromolecule-associated signal transduction,” and “signal transduction.” Among these clusters, the “positive regulation of metabolic process” cluster exhibited the highest values across all subtypes, indicating the strongest association with increased risk. GFRA1, identified as the key protein in this cluster, had its significance validated through DEA. Moreover, MMP12 and DKK4, the most critical proteins in the other two clusters, have been confirmed as plasma biomarkers significantly associated with dementia in various studies [16, 63, 64]. These findings align closely with existing clinical research, underscoring the robustness of our results.

## Discussion

This study aimed to predict the neuropathological subtypes of dementia with plasma biomarkers by developing a novel ML model. We designed NeuroFANN to be further biologically informed by reflecting the interactive and functional properties of proteins. Our model utilized the PPI-based synergetic effects of proteins, encompassing the collective contribution of multiple proteins to the neuropathological subtypes, in addition to the functional annotation-based clustering of biomarkers, considering the balanced and diversified biological functions of proteins. To the best of our knowledge, this is the first attempt to simultaneously implement those two components in a single ML model, while they have been applied to ML separately in previous studies [31, 65, 66].

NeuroFANN outperformed seven GNN-based models in prediction for all three subtypes, even when it only applied the network propagation, with a significantly enhanced performance compared to that of training the independent effects of biomarkers, suggesting the validity of utilizing the PPI-based synergetic effects. The performance comparison results also implicate the necessity of utilizing the PPI in a global range. The network propagation of our model enabled reflecting interactions between distant proteins in the PPI network, while the comparison models only reflected the interactions between neighboring proteins within a local range: 2-hop for GCN and SGC, 3-hop for EGC, 4-hop for MixHop and UGCN, 5-hop for LGC, and 6-hop for MOGCN, indicating the performance of comparison models tended to be proportional to the number of hops.

This study enabled understanding the contribution of functionally clustered biomarkers to the neuropathological subtypes, covering metabolic function [55, 67, 68], neuron development [69], extracellular region [70, 71], immune response [72], and cellular communication [73]. We identified the “positive regulation of metabolic process” as the most important biomarker cluster for predicting neuropathological subtypes. The most informative proteins in this cluster were GFRA1 and NTRK3, consistent with the DEP analysis results indicating that these were the remarkably upregulated proteins for the neuropathological subtypes, including clinical implications revealed that GFRA1 and NTRK3 are significantly associated with neurodegenerative changes [74–77], binding with the nerve growth factor or the glial cell line-derived neurotrophic factor that are related to metabolic dysfunction [78–80]. Our findings also covered well-known dementia-associated proteins, such as BCAN, CNTN5, and NCAN [81, 82], along with identifying recently promising biomarkers, including IL18, MMP12, TNFSF12, and UNC5C [63, 83–85], where these biomarkers presented the highest protein importance in their clusters.

### Limitations

This study has limitations as follows. First, the plasma biomarker identification was limited to assayed proteins included in the neurology and cytokine panels. Although both panels yielded valuable biomarkers for neuropathological subtypes, this study should be extended to utilize the additional panels to gain a more comprehensive understanding of dementia’s biological nature. Second, this study did not consider tauopathy to be a neuropathological subtype due to the absence of tau PET scans for the participants. Since tauopathy is an important factor in dementia, our model should be complemented to include it. Third, the findings in this study were limited to the single cohort for the Korean and to the internal validation. Therefore, it is challenging to ensure that our model will perform consistently well across different cohorts with various ethnicities, requiring a further demonstration of the model using the external validation datasets.

## Conclusion

In this diagnostic study on neuropathological subtypes of dementia, we developed a predictive ML model that reflects the in-depth biological properties of plasma biomarkers. By identifying PPI-based synergetic effects, clustering biomarkers based on functional annotations, and utilizing them in the ML model, we gained a deeper understanding of the molecular nature of plasma biomarkers, leading to more accurate prediction. Our findings suggest that the developed model can contribute to the clinical field as a diagnostic aid for more precise screening of patients with dementia.

**Key Points**

- We propose NeuroFANN, a novel machine learning model designed to identify neuropathological subtypes in dementia—positivity for amyloid beta deposition, medial temporal lobe atrophy, and white matter hyperintensity—using plasma protein biomarkers.
- NeuroFANN provides biologically informed risk predictions for dementia subtypes by incorporating the protein–protein interaction (PPI) network and functional annotation of protein biomarkers.
- By integrating the PPI network with independent effects of proteins, NeuroFANN captures synergetic effects through globality-based feature aggregation, effectively representing the complex properties of PPIs.
- NeuroFANN employs functional annotation-based protein clustering, enabling a biologically meaningful and balanced analysis of dementia-related processes.
- Experimental validation demonstrates NeuroFANN’s superior classification performance, showcasing its ability to identify biologically relevant proteins and generate interpretable results.

## Supplementary Material

NeuroFANN_Supplement_bbaf366

## Data Availability

The data used in this study are available upon request from the BICWALZS consortium biobank (http://www.bicwalzs.com) as well as the National Biobank of Korea (https://biobank.nih.go.kr) which is a central biobank supporting for the Korea Biobank Project. The source codes of this study are available at https://github.com/sunghongpark-ai/NeuroFANN.
