# Supplementary material for: NeuroFANN: identification of neuropathological subtypes in dementia with plasma proteins by using functionally annotated neural network
Source: Brief Bioinform. 2025 Aug 1;26(4):bbaf366. doi: 10.1093/bib/bbaf366 (PMC12315549; doi:10.1093/bib/bbaf366)
Supplement: NeuroFANN_Supplement_bbaf366 [file neurofann_supplement_bbaf366.docx]

Supplementary Data

NeuroFANN: identification of neuropathological subtypes in dementia with plasma proteins by using functionally annotated neural network

Sunghong Park^1^^,^^†^, Doyoon Kim^1,†^, Ji-Hye Choi^1^, Chang Hyung Hong^2^, Sang Joon Son^2^, Hyun Woong Roh^2^, Hyunjung Shin^3,4,*^, Hyun Goo Woo^1,5,6,*^

^1^Department of Physiology, Ajou University School of Medicine, Suwon, 16499, Republic of Korea

^2^Department of Psychiatry, Ajou University School of Medicine, Suwon, 16499, Republic of Korea

^3^Department of Industrial Engineering, Ajou University, Suwon, 16499, Republic of Korea

^4^Department of Artificial Intelligence, Ajou University, Suwon, 16499, Republic of Korea

^5^Department of Biomedical Science, Graduate School of Ajou University, Suwon, 16499, Republic of Korea

^6^Ajou Translational Omics Center, Research Institute for Innovative Medicine, Ajou University Medical Center, Suwon, 16499, Republic of Korea

^†^ These authors contributed equally to this work.

**^*^ Corresponding authors:** Hyunjung Shin ([shin@ajou.ac.kr](mailto:shin@ajou.ac.kr)) and Hyun Goo Woo ([hg@ajou.ac.kr](mailto:hg@ajou.ac.kr))

**Contents**

**Supplementary Table S1.** List of assayed proteins in the Olink panels

**Supplementary Table S2.** Differentially expressed proteins for neuropathological dementia subtypes

**Supplementary Table S3.** Biomarker clusters and the corresponding proteins

**Supplementary Table S4.** Average performance evaluations of baseline models

**Supplementary Table S5.** Average performance evaluations of ablated models

**Supplementary Table S6.** Model-driven protein importances for entire plasma biomarkers

**Supplementary Table S1.** List of assayed proteins in the Olink panels

| (a) Assayed proteins in the Olink Target 96 Neurology panel | | | | | | | | |
| --- | --- | --- | --- | --- | --- | --- | --- | --- |
| No. | Protein Name | Uniprot ID | No. | Protein Name | Uniprot ID | No. | Protein Name | Uniprot ID |
| 1 | ACVRL1 | P37023 | 32 | FCRL2 | Q96LA5 | 63 | NRCAM | Q92823 |
| 2 | ADAM22 | Q9P0K1 | 33 | FLRT2 | O43155 | 64 | NRP2 | O60462 |
| 3 | ADAM23 | O75077 | 34 | FRZB | Q92765 | 65 | NTRK2 | Q16620 |
| 4 | ASAH2 | Q9NR71 | 35 | GDNF | P39905 | 66 | NTRK3 | Q16288 |
| 5 | BCAN | Q96GW7 | 36 | GFRA1 | P56159 | 67 | PDGFRA | P16234 |
| 6 | BMP4 | P12644 | 37 | GFRA3 | O60609 | 68 | PLXNB1 | O43157 |
| 7 | CADM3 | Q8N126 | 38 | GPC5 | P78333 | 69 | PLXNB3 | Q9ULL4 |
| 8 | CD200 | P41217 | 39 | GZMA | P12544 | 70 | PRTG | Q2VWP7 |
| 9 | CD200R1 | Q8TD46 | 40 | HAGH | Q16775 | 71 | PVR | P15151 |
| 10 | CD300C | Q08708 | 41 | IL12A | P29460 | 72 | RGMA | Q96B86 |
| 11 | CD300LF | Q8TDQ1 | 42 | IL5RA | Q01344 | 73 | RGMB | Q6NW40 |
| 12 | CD38 | P28907 | 43 | JAM2 | P57087 | 74 | ROBO2 | Q9HCK4 |
| 13 | CDH3 | P22223 | 44 | KYNU | Q16719 | 75 | RSPO1 | Q2MKA7 |
| 14 | CDH6 | P55285 | 45 | LAIR2 | Q6ISS4 | 76 | SCARA5 | Q6ZMJ2 |
| 15 | CLEC10A | Q8IUN9 | 46 | LAT | O43561 | 77 | SCARB2 | Q14108 |
| 16 | CLEC1B | Q9P126 | 47 | LAYN | Q6UX15 | 78 | SCARF2 | Q96GP6 |
| 17 | CNTN5 | O94779 | 48 | LGALS8 | O00214 | 79 | SIGLEC1 | Q9BZZ2 |
| 18 | CPA2 | P48052 | 49 | LRPAP1 | P30533 | 80 | SIGLEC9 | Q9Y336 |
| 19 | CPM | P14384 | 50 | LXN | Q9BS40 | 81 | SMOC2 | Q9H3U7 |
| 20 | CRTAM | O95727 | 51 | MANF | P55145 | 82 | SMPD1 | P17405 |
| 21 | CSF2RA | P15509 | 52 | MAPT | P10636 | 83 | SPOCK1 | Q08629 |
| 22 | CSF3 | P09919 | 53 | MATN3 | O15232 | 84 | THY1 | P04216 |
| 23 | CTSC | P53634 | 54 | MDGA1 | Q8NFP4 | 85 | TMPRSS5 | Q9H3S3 |
| 24 | CTSS | P25774 | 55 | MME | P08473 | 86 | TNFRSF12A | Q9NP84 |
| 25 | DDR1 | Q08345 | 56 | MSR1 | P21757 | 87 | TNFRSF21 | O75509 |
| 26 | DKK4 | Q9UBT3 | 57 | MSTN | O14793 | 88 | TNR | Q92752 |
| 27 | DRAXIN | Q8NBI3 | 58 | NAAA | Q02083 | 89 | ULBP2 | Q9BZM5 |
| 28 | EDA2R | Q9HAV5 | 59 | NBL1 | P41271 | 90 | UNC5C | O95185 |
| 29 | EFNA4 | P52798 | 60 | NCAN | O14594 | 91 | VWC2 | Q2TAL6 |
| 30 | EPHB6 | O15197 | 61 | NGF | P01138 | 92 | WFIKKN1 | Q96NZ8 |
| 31 | EZR | P15311 | 62 | NMNAT1 | Q9HAN9 |  |  |  |
| (b) Assayed proteins in the Olink Target 48 Cytokine panel | | | | | | | | |
| No. | Protein Name | Uniprot ID | No. | Protein Name | Uniprot ID | No. | Protein Name | Uniprot ID |
| 1 | CCL11 | P51671 | 16 | CXCL9 | Q07325 | 31 | IL33 | O95760 |
| 2 | CCL13 | Q99616 | 17 | EGF | P01133 | 32 | IL4 | P05112 |
| 3 | CCL19 | Q99731 | 18 | FLT3LG | P49771 | 33 | IL6 | P05231 |
| 4 | CCL2 | P13500 | 19 | HGF | P14210 | 34 | IL7 | P13232 |
| 5 | CCL3 | P10147 | 20 | IFNG | P01579 | 35 | LTA | P01374 |
| 6 | CCL4 | P13236 | 21 | IL10 | P22301 | 36 | MMP1 | P03956 |
| 7 | CCL7 | P80098 | 22 | IL13 | P35225 | 37 | MMP12 | P39900 |
| 8 | CCL8 | P80075 | 23 | IL15 | P40933 | 38 | OLR1 | P78380 |
| 9 | CSF1 | P09603 | 24 | IL17A | Q16552 | 39 | OSM | P13725 |
| 10 | CSF2 | P04141 | 25 | IL17C | Q9P0M4 | 40 | TGFA | P01135 |
| 11 | CSF3 | P09919 | 26 | IL17F | Q96PD4 | 41 | TNF | P01375 |
| 12 | CXCL10 | P02778 | 27 | IL18 | Q14116 | 42 | TNFSF10 | P50591 |
| 13 | CXCL11 | O14625 | 28 | IL1B | P01584 | 43 | TNFSF12 | O43508 |
| 14 | CXCL12 | P48061 | 29 | IL2 | P60568 | 44 | TSLP | Q969D9 |
| 15 | CXCL8 | P10145 | 30 | IL27 | Q8NEV9 | 45 | VEGFA | P15692 |

**Supplementary Table S2.** Differentially expressed proteins for neuropathological dementia subtypes

| (a) Differentially expressed proteins for Aβ | | | | | | | |
| --- | --- | --- | --- | --- | --- | --- | --- |
| Protein | log_2_FC | *P*-value | Regulation | Protein | log_2_FC | *P*-value | Regulation |
| NTRK3 | +0.151 | 0.021 | UP | FCRL2 | –0.172 | 0.011 | DOWN |
| IL33 | +0.140 | 0.037 | UP | TNF | –0.175 | 0.009 | DOWN |
| NCAN | –0.138 | 0.035 | DOWN | CNTN5 | –0.177 | 0.007 | DOWN |
| ROBO2 | –0.141 | 0.035 | DOWN | BCAN | –0.189 | 0.004 | DOWN |
| CD300LF | –0.150 | 0.024 | DOWN | CCL19 | –0.190 | 0.007 | DOWN |
| (b) Differentially expressed proteins for MTA | | | | | | | |
| Protein | log_2_FC | *P*-value | Regulation | Protein | log_2_FC | *P*-value | Regulation |
| GFRA1 | +0.259 | < 0.001 | UP | SMOC2 | +0.173 | 0.012 | UP |
| SMPD1 | +0.252 | < 0.001 | UP | IL27 | +0.163 | 0.019 | UP |
| EDA2R | +0.249 | < 0.001 | UP | TNFRSF12A | +0.162 | 0.018 | UP |
| MMP12 | +0.228 | 0.001 | UP | EFNA4 | +0.160 | 0.019 | UP |
| CCL13 | +0.225 | 0.002 | UP | EPHB6 | +0.150 | 0.031 | UP |
| MSR1 | +0.204 | 0.003 | UP | LAT | +0.145 | 0.042 | UP |
| PLXNB3 | +0.201 | 0.004 | UP | CTSC | +0.142 | 0.047 | UP |
| CCL11 | +0.198 | 0.004 | UP | LAYN | +0.140 | 0.043 | UP |
| DKK4 | +0.194 | 0.004 | UP | PVR | +0.135 | 0.048 | UP |
| ACVRL1 | +0.191 | 0.006 | UP | CNTN5 | –0.161 | 0.025 | DOWN |
| SCARF2 | +0.185 | 0.007 | UP | BCAN | –0.285 | < 0.001 | DOWN |
| CCL3 | +0.177 | 0.011 | UP |  |  |  |  |
| (c) Differentially expressed proteins for WMH | | | | | | | |
| Protein | log_2_FC | *P*-value | Regulation | Protein | log_2_FC | *P*-value | Regulation |
| GFRA1 | +0.236 | < 0.001 | UP | NRP2 | +0.142 | 0.018 | UP |
| MMP12 | +0.219 | < 0.001 | UP | ULBP2 | +0.141 | 0.018 | UP |
| MSR1 | +0.212 | < 0.001 | UP | CCL11 | +0.141 | 0.021 | UP |
| DKK4 | +0.184 | 0.002 | UP | FLRT2 | +0.138 | 0.017 | UP |
| EPHB6 | +0.184 | 0.002 | UP | SCARA5 | +0.138 | 0.019 | UP |
| SMPD1 | +0.177 | 0.004 | UP | CSF1 | +0.138 | 0.023 | UP |
| SCARF2 | +0.177 | 0.003 | UP | PVR | +0.137 | 0.021 | UP |
| PLXNB3 | +0.174 | 0.004 | UP | CDH3 | +0.135 | 0.025 | UP |
| TNFRSF21 | +0.172 | 0.004 | UP | IL12A | +0.135 | 0.027 | UP |
| CLEC10A | +0.170 | 0.005 | UP | IL15 | +0.134 | 0.026 | UP |
| SCARB2 | +0.170 | 0.004 | UP | SMOC2 | +0.133 | 0.026 | UP |
| NTRK3 | +0.166 | 0.007 | UP | CD300C | +0.133 | 0.027 | UP |
| EFNA4 | +0.162 | 0.006 | UP | UNC5C | +0.126 | 0.033 | UP |
| IL27 | +0.162 | 0.007 | UP | CCL3 | +0.126 | 0.040 | UP |
| CCL13 | +0.161 | 0.010 | UP | ADAM23 | +0.126 | 0.036 | UP |
| CXCL9 | +0.155 | 0.010 | UP | IL17C | +0.124 | 0.042 | UP |
| EDA2R | +0.154 | 0.015 | UP | ADAM22 | +0.123 | 0.043 | UP |
| RGMB | +0.154 | 0.012 | UP | LAYN | +0.121 | 0.042 | UP |
| ACVRL1 | +0.153 | 0.012 | UP | TNFSF12 | –0.114 | 0.046 | DOWN |
| VWC2 | +0.153 | 0.010 | UP | CNTN5 | –0.129 | 0.037 | DOWN |
| RSPO1 | +0.152 | 0.010 | UP | IL1B | –0.143 | 0.020 | DOWN |
| CTSC | +0.146 | 0.016 | UP | BCAN | –0.150 | 0.013 | DOWN |
| TNFRSF12A | +0.144 | 0.016 | UP | IL18 | –0.178 | 0.004 | DOWN |

**Supplementary Table S3.** Biomarker clusters and the corresponding proteins

| No. | Biomarker cluster | Protein | No. | Biomarker cluster | Protein |
| --- | --- | --- | --- | --- | --- |
| 1 | Chemokines | CCL11 | 9 | Molecular transducer activity | CD300C |
|  |  | CCL13 |  |  | CLEC10A |
|  |  | CXCL9 |  |  | NCAN |
| 2 | Cytokine associated signal transduction | CD300LF |  |  | SCARB2 |
|  |  | EDA2R | 10 | Neuron development associated  cell motility | FLRT2 |
| 3 | Cytokines | CCL19 |  |  | NRP2 |
|  |  | CCL3 |  |  | PLXNB3 |
|  |  | CSF1 |  |  | UNC5C |
|  |  | IL12A | 11 | Neuron development associated  signal trasnduction | CNTN5 |
|  |  | IL15 |  |  | EFNA4 |
|  |  | IL18 |  |  | EPHB6 |
|  |  | IL1B |  |  | TNFRSF21 |
|  |  | IL33 | 12 | Positive regulation of adaptive  immune response | PVR |
|  |  | TNF |  |  | ULBP2 |
| 4 | Extracellular matrix | BCAN | 13 | Positive regulation of metabolic process | GFRA1 |
|  |  | VWC2 |  |  | LAT |
| 5 | Focal adhesion | LAYN |  |  | NTRK3 |
|  |  | SCARF2 |  |  | RSPO1 |
| 6 | Leukocyte activation | CTSC |  |  | SMPD1 |
|  |  | IL27 | 14 | Programmed cell death | ROBO2 |
|  |  | TNFSF12 |  |  | TNFRSF12A |
| 7 | Macromolecule associated  signal transduction | ACVRL1 | 15 | Scavenger receptor type A | MSR1 |
|  |  | CDH3 |  |  | SCARA5 |
|  |  | MMP12 | 16 | Signal transduction | ADAM22 |
|  |  | SMOC2 |  |  | ADAM23 |
| 8 | Macromolecule associated  signaling receptor | FCRL2 |  |  | DKK4 |
|  |  | RGMB |  |  | IL17C |

**Supplementary Table S4.** Average performance evaluations of baseline models

| Model | Average performance (mean [SD] 95% CI)^*^ | | | | | | Overall average |
| --- | --- | --- | --- | --- | --- | --- | --- |
|  | AUROC | Accuracy | Sensitivity | Specificity | PPV | NPV |  |
| NeuroFANN  (Ours) | 0.832 [0.007]  (0.830–0.833) | 0.763 [0.015]  (0.760–0.766) | 0.765 [0.046]  (0.756–0.774) | 0.750 [0.038]  (0.742–0.757) | 0.583 [0.030]  (0.577–0.588) | 0.886 [0.016]  (0.883–0.889) | 0.763 [0.005]  (0.762–0.764) |
| GCN | 0.739 [0.002]  (0.739–0.739) | 0.678 [0.011]  (0.676–0.680) | 0.804 [0.027]  (0.799–0.810) | 0.610 [0.025]  (0.605–0.615) | 0.477 [0.011]  (0.475–0.479) | 0.881 [0.010]  (0.879–0.883) | 0.698 [0.003]  (0.698–0.699) |
| SGC | 0.766 [0.002]  (0.766–0.767) | 0.701 [0.020]  (0.697–0.705) | 0.797 [0.049]  (0.788–0.807) | 0.636 [0.039]  (0.629–0.644) | 0.500 [0.020]  (0.496–0.503) | 0.893 [0.014]  (0.890–0.895) | 0.715 [0.003]  (0.715–0.716) |
| EGC | 0.768 [0.002]  (0.768–0.769) | 0.738 [0.017]  (0.735–0.741) | 0.734 [0.039]  (0.726–0.742) | 0.705 [0.031]  (0.698–0.711) | 0.552 [0.024]  (0.547–0.557) | 0.882 [0.010]  (0.880–0.884) | 0.730 [0.004]  (0.729–0.731) |
| LGC | 0.773 [0.002]  (0.772–0.773) | 0.743 [0.015]  (0.740–0.746) | 0.733 [0.034]  (0.726–0.739) | 0.713 [0.028]  (0.708–0.719) | 0.554 [0.021]  (0.550–0.558) | 0.881 [0.008]  (0.879–0.883) | 0.733 [0.004]  (0.732–0.734) |
| MixHop | 0.788 [0.001]  (0.787–0.788) | 0.722 [0.012]  (0.719–0.724) | 0.787 [0.028]  (0.782–0.793) | 0.688 [0.025]  (0.683–0.692) | 0.525 [0.010]  (0.523–0.527) | 0.884 [0.009]  (0.882–0.886) | 0.732 [0.002]  (0.732–0.733) |
| UGCN | 0.798 [0.003]  (0.798–0.799) | 0.728 [0.010]  (0.726–0.730) | 0.793 [0.031]  (0.788–0.801) | 0.691 [0.030]  (0.685–0.697) | 0.529 [0.016]  (0.526–0.533) | 0.889 [0.013]  (0.886–0.891) | 0.738 [0.003]  (0.738–0.738) |
| MOGCN | 0.800 [0.003]  (0.799–0.801) | 0.731 [0.013]  (0.729–0.734) | 0.784 [0.034]  (0.778–0.791) | 0.700 [0.030]  (0.694–0.705) | 0.534 [0.018]  (0.531–0.538) | 0.886 [0.011]  (0.884–0.889) | 0.739 [0.004]  (0.739–0.740) |

Abbreviations: AUROC, area under the receiver operating characteristics curve; EGC, exponential graph convolution; GCN, graph convolutional network; LGC, linear graph convolution; MOGCN, mixed-order graph convolutional network; NPV, negative predictive value; PPV, positive predictive value; SGC, simple graph convolution; UGCN, universal graph convolutional network.

^*^Each model underwent 100 repeated training, with 20 iterations of 5-fold cross-validation. The resulting performances of each model were then listed, and the mean was calculated.

**Supplementary Table S5.** Average performance evaluations of ablated models

| Model | Average performance (mean [SD] 95% CI)^*^ | | | | | | Overall average |
| --- | --- | --- | --- | --- | --- | --- | --- |
|  | AUROC | Accuracy | Sensitivity | Specificity | PPV | NPV |  |
| Ablated  Model 1^a^ | 0.817 [0.010]  (0.814–0.819) | 0.732 [0.022]  (0.728–0.737) | 0.787 [0.059]  (0.775–0.798) | 0.695 [0.055]  (0.684–0.706) | 0.540 [0.030]  (0.534–0.546) | 0.891 [0.023]  (0.886–0.895) | 0.744 [0.008]  (0.742–0.745) |
| Ablated  Model 2^b^ | 0.806 [0.007]  (0.805–0.808) | 0.731 [0.023]  (0.727–0.736) | 0.771 [0.057]  (0.759–0.782) | 0.703 [0.048]  (0.694–0.713) | 0.541 [0.030]  (0.535–0.547) | 0.884 [0.017]  (0.880–0.887) | 0.739 [0.006]  (0.738–0.740) |
| Ablated  Model 3^c^ | 0.769 [0.010]  (0.767–0.771) | 0.670 [0.025]  (0.665–0.675) | 0.787 [0.053]  (0.776–0.797) | 0.602 [0.059]  (0.590–0.614) | 0.478 [0.024]  (0.473–0.483) | 0.876 [0.023]  (0.872–0.881) | 0.697 [0.009]  (0.695–0.699) |
| Ablated  Model 4^d^ | 0.688 [0.002]  (0.688–0.688) | 0.628 [0.006]  (0.627–0.629) | 0.807 [0.013]  (0.805–0.810) | 0.520 [0.014]  (0.517–0.523) | 0.442 [0.004]  (0.441–0.443) | 0.885 [0.009]  (0.883–0.886) | 0.662 [0.002]  (0.661–0.662) |

Abbreviations: AUROC, area under the receiver operating characteristics curve; NPV, negative predictive value; PPV, positive predictive value.

^*^Each model underwent 100 repeated training, with 20 iterations of 5-fold cross-validation. The resulting performances of each model were then listed, and the mean was calculated.

^a^This model consisted of the network propagation of the developed model and the equal aggregation of the synergetic effects of plasma biomarkers.

^b^This model was configured by ablating the cluster aggregation from the developed model.

^c^This model was configured by ablating the network propagation from the developed model.

^d^This model was configured by ablating both network propagation and cluster aggregation from the developed model.

**Supplementary Table S6.** Model-driven protein importances for entire plasma biomarkers

| Protein | Importance (mean [SD] 95% CI) | Protein | Importance (mean [SD] 95% CI) |
| --- | --- | --- | --- |
| *Positive regulation of metabolic process* | | *Leukocyte activation* | |
| GFRA1 | 0.366 [0.208] (0.324–0.407) | TNFSF12 | 0.806 [0.180] (0.771–0.842) |
| NTRK3 | 0.220 [0.142] (0.192–0.248) | CTSC | 0.133 [0.123] (0.109–0.158) |
| LAT | 0.191 [0.335] (0.124–0.257) | IL27 | 0.060 [0.077] (0.045–0.076) |
| RSPO1 | 0.177 [0.095] (0.158–0.196) | *Chemokines* | |
| SMPD1 | 0.047 [0.041] (0.039–0.055) | CXCL9 | 0.373 [0.342] (0.305–0.441) |
| *Extracellular matrix* | | CCL13 | 0.340 [0.350] (0.271–0.410) |
| BCAN | 0.918 [0.189] (0.881–0.956) | CCL11 | 0.286 [0.326] (0.221–0.351) |
| VWC2 | 0.082 [0.189] (0.044–0.119) | *Cytokines* | |
| *Neuron development associated signal transduction* | | IL18 | 0.337 [0.270] (0.283–0.390) |
| CNTN5 | 0.398 [0.260] (0.346–0.449) | CCL19 | 0.163 [0.293] (0.105–0.221) |
| TNFRSF21 | 0.323 [0.251] (0.273–0.373) | IL33 | 0.162 [0.181] (0.126–0.198) |
| EPHB6 | 0.204 [0.168] (0.170–0.237) | IL15 | 0.106 [0.128] (0.080–0.131) |
| EFNA4 | 0.075 [0.096] (0.056–0.095) | IL1B | 0.100 [0.140] (0.072–0.128) |
| *Signal transduction* | | TNF | 0.054 [0.098] (0.035–0.074) |
| DKK4 | 0.542 [0.418] (0.459–0.625) | IL12A | 0.029 [0.078] (0.013–0.044) |
| IL17C | 0.272 [0.297] (0.213–0.331) | CSF1 | 0.028 [0.038] (0.021–0.036) |
| ADAM22 | 0.096 [0.148] (0.067–0.126) | CCL3 | 0.021 [0.030] (0.015–0.027) |
| ADAM23 | 0.089 [0.135] (0.063–0.116) | *Macromolecule associated signaling receptor* | |
| *Molecular transducer activity* | | FCRL2 | 0.586 [0.388] (0.509–0.663) |
| NCAN | 0.488 [0.177] (0.453–0.523) | RGMB | 0.414 [0.388] (0.337–0.491) |
| CLEC10A | 0.246 [0.122] (0.222–0.270) | *Macromolecule associated signal transduction* | |
| CD300C | 0.184 [0.104] (0.163–0.204) | MMP12 | 0.502 [0.304] (0.441–0.562) |
| SCARB2 | 0.082 [0.095] (0.063–0.101) | ACVRL1 | 0.185 [0.192] (0.147–0.223) |
| *Cytokine associated signal transduction* | | SMOC2 | 0.179 [0.183] (0.143–0.215) |
| CD300LF | 0.848 [0.182] (0.812–0.884) | CDH3 | 0.134 [0.186] (0.097–0.171) |
| EDA2R | 0.152 [0.182] (0.116–0.188) | *Positive regulation of adaptive immune response* | |
| *Neuron development associated cell motility* | | PVR | 0.568 [0.264] (0.515–0.620) |
| UNC5C | 0.549 [0.347] (0.480–0.618) | ULBP2 | 0.432 [0.264] (0.380–0.485) |
| FLRT2 | 0.156 [0.164] (0.124–0.189) | *Scavenger receptor type A* | |
| NRP2 | 0.150 [0.138] (0.122–0.177) | MSR1 | 0.822 [0.148] (0.793–0.851) |
| PLXNB3 | 0.145 [0.194] (0.107–0.183) | SCARA5 | 0.178 [0.148] (0.149–0.207) |
| *Programmed cell death* | | *Focal adhesion* | |
| ROBO2 | 0.782 [0.176] (0.747–0.817) | SCARF2 | 0.564 [0.352] (0.494–0.634) |
| TNFRSF12A | 0.218 [0.176] (0.183–0.253) | LAYN | 0.436 [0.352] (0.366–0.506) |
